# Balloon Delivery Difficulties during Endoscopic Balloon Dilation for Crohn’s Disease-Associated Strictures

**DOI:** 10.1159/iid/adeag002

**Published:** 2026-08-10

**Authors:** Tomohiro Fukuda, Yuichi Morohoshi, Kohei Morioka, Yuya Hagihara, Yuya Tsunoda, Satoshi Imamura, Shuichi Nagakubo, Yuji Koike, Yuriko Fujita

**Affiliations:** Department of Gastroenterology, Yokohama Municipal Citizen’s Hospital, Yokohama, Japan

**Keywords:** Crohn’s disease, Endoscopic balloon dilation, Cohort study

## Abstract

**Introduction:**

Endoscopic balloon dilation (EBD) is an established and effective treatment for gastrointestinal strictures associated with Crohn’s disease (CD). However, technical complications related to balloon delivery have not been systematically evaluated. This study aimed to assess the incidence, characteristics, and risk factors associated with balloon delivery difficulties (BDDs) during EBD.

**Methods:**

This retrospective, single-center study analyzed EBD sessions performed for gastrointestinal strictures associated with CD. Each EBD session was considered the unit of analysis. BDD was defined as difficulty in balloon withdrawal, balloon insertion, or balloon damage. The session-based incidence of BDD and associated factors were evaluated.

**Results:**

A total of 319 EBD sessions were included. BDD occurred in 33 sessions (10.3%). The types of BDD included difficulty in balloon withdrawal (11 events), difficulty in balloon insertion (24 events), and balloon damage (6 events). Six BDD events occurred during the first EBD attempt within a session, whereas 27 events occurred during the second and subsequent attempts. Difficulty in balloon insertion predominantly occurred during repeated EBD attempts. A higher number of EBD attempts within the same session (odds ratio [OR], 2.56; 95% confidence interval [CI], 1.07–6.80; *p* = 0.025) and a smaller working channel diameter (OR, 3.13; 95% CI, 1.28–7.69; *p* = 0.009) were associated with BDD.

**Conclusion:**

BDD occurred in approximately 10% of EBD sessions and was more likely to develop during repeated dilation attempts within the same session. These findings may help inform endoscopists about procedural risk and optimize dilation strategies during EBD.

## Plain Language Summary

Crohn’s disease can cause narrowing (strictures) in the intestine, which may lead to symptoms such as abdominal pain or bowel obstruction. Endoscopic balloon dilation (EBD) is a procedure used to widen these narrowed areas without surgery. In this study, we investigated technical problems that can occur during balloon delivery in EBD. These problems, called balloon delivery difficulties (BDDs), include difficulty in inserting or withdrawing the balloon and damage to the balloon. BDD may interrupt the procedure, making it difficult to complete the planned dilation or to reach more proximal segments of the intestine. We analyzed 319 EBD procedures and found that BDD occurred in about 10% of procedures. They were more likely to occur when balloon dilation was repeated within the same procedure. Our findings suggest that recognizing these technical difficulties may help optimize procedural strategies, improve the efficiency of EBD, and facilitate successful completion of the planned procedure.

## Introduction

Crohn’s disease (CD) is a chronic relapsing inflammatory disorder that frequently progresses to luminal narrowing and a fibrostenotic phenotype over the course of the disease [1, 2]. Intestinal strictures are a major cause of obstructive symptoms and often necessitate surgical intervention [3]. Endoscopic balloon dilation (EBD) is an effective bowel-preserving therapeutic strategy for small bowel strictures associated with CD [4–6].

In recent years, the efficacy and safety of EBD have also been reported in patients with multiple strictures [7, 8]. In such cases, dilation often needs to be performed repeatedly during a single endoscopic session, requiring multiple insertions and withdrawals of the same dilation balloon catheter. During this process, the balloon is repeatedly delivered through the endoscope working channel, which may compromise the reliability of balloon delivery. Incomplete balloon deflation or deformation may increase resistance during insertion and adversely affect maneuverability.

Furthermore, balloon delivery difficulties (BDDs), such as difficulty in balloon withdrawal due to incomplete deflation, difficulty in balloon insertion, and balloon damage, may occur during EBD procedures. Although these events are not necessarily severe, they may disrupt the procedural flow and hinder repeated dilation attempts. Failure to successfully traverse a stricture in patients with multiple strictures may preclude the evaluation and treatment of proximal lesions. Therefore, BDD is clinically relevant in daily endoscopic practice.

However, the incidence and detailed characteristics of BDD have not been systematically investigated. Balloon delivery is generally regarded as an ancillary procedural step rather than an independent subject of evaluation, and data specifically focusing on these technical issues remain scarce. Therefore, the present study aimed to investigate the incidence and characteristics of BDD during EBD for CD.

## Methods

### Study Design and Participants

This was a single-center, retrospective, observational study. Patients who underwent EBD for CD-associated strictures at our institution between January 2017 and May 2025 were enrolled. All procedures were performed using enteroscopes and both antegrade and retrograde approaches were permitted. Both short- and long-type enteroscopes, including single- and double-balloon enteroscopes, were used. The stricture location was not restricted and included the anus, colon, small intestine, and postoperative anastomosis. The unit of analysis in this study was an individual EBD session, defined as a single endoscopic examination during which ≥1 EBD procedures were performed. Multiple sessions with the same patient were permitted if the sessions were performed on different dates. The number of EBD procedures per session was defined as the total number of procedures performed in a single session. Each dilation was referred to as an EBD attempt, and the number of attempts (first, second, or subsequent) was recorded to evaluate the timing of BDD occurrence.

### EBD Procedures

EBD was performed as part of routine clinical practice by experienced endoscopists specializing in IBD-related endoscopic interventions. All procedures were performed using balloon-assisted enteroscopes from Olympus Medical Systems (Tokyo, Japan) and Fujifilm Corporation (Tokyo, Japan), equipped with working channel diameters of 2.8–3.2 mm. Through-the-scope balloon dilators, including EZDilate (Olympus Medical Systems, Tokyo, Japan), Elation 5-Stage Fixed-Wire Dilation Balloon (Merit Medical Systems, Inc., South Jordan, UT, USA), and Hercules 3 Stage Wire-Guided Balloon (Cook Medical, Bloomington, IN, USA), were available and selected according to standard clinical practice. The type of dilation balloon, target dilation diameter, inflation pressure, and number of dilations were determined at the discretion of the endoscopist based on the location and characteristics of the stricture. During EBD, olive oil was injected into the working channel to improve lubrication before the dilation balloon catheter was inserted. After securing the proximal bowel lumen with a guide wire, EBD was performed under wire guidance.

### Outcomes and Definitions

The primary outcome measure was the occurrence of BDD during EBD. BDD was defined as the occurrence of any of the following events during EBD: difficulty in balloon withdrawal, difficulty in balloon insertion, or balloon damage. BDD was evaluated on a per-session basis, and sessions in which ≥1 BDD event occurred were classified as “with BDD.” Difficulty in balloon withdrawal was defined as the inability to remove the balloon catheter using standard traction, which required withdrawal along with the endoscope. Difficulty in balloon insertion was defined as failure to insert the balloon catheter through the working channel using standard techniques, necessitating the use of a new balloon catheter or abandonment of further dilation. Balloon damage was defined as physical damage to the balloon catheter that was explicitly documented in endoscopic reports or medical records.

The secondary outcomes included a breakdown of BDD events (difficulty in balloon withdrawal and insertion, and balloon damage) and the occurrence of EBD-related complications. All outcomes were retrospectively assessed based on endoscopic reports and medical records. In addition, potential risk factors for BDD were evaluated, including the working channel diameter, balloon diameter, scope length, number of EBD attempts performed within the same session, stricture location, and presence of postoperative anastomosis.

### Covariates

Data on sex, age, disease duration, disease location, number of strictures, stricture location, number of EBD procedures per session, balloon diameter, and occurrence of BDD events, including difficulty in balloon insertion, difficulty in balloon withdrawal, and balloon damage, were collected. The number of attempts at which BDD occurred in each EBD session was also recorded.

### Statistical Analysis

The baseline patient characteristics and endoscopic procedural factors were summarized using descriptive statistics. Continuous variables were presented as medians with interquartile ranges or means with standard deviations, as appropriate, and categorical variables were presented as absolute numbers with percentages. The overall proportion of BDD and their distribution according to the BDD type were calculated.

The primary unit of analysis was individual EBD sessions. Accordingly, analyses of the incidence and risk factors of BDD were performed at the session level. By contrast, analyses evaluating the timing of BDD occurrence were performed at the individual EBD attempt level and were intended to be descriptive assessments of the temporal distribution of BDD events within a session.

Univariate comparisons of the baseline and procedural factors were performed between sessions with and without BDD as an exploratory analysis. Categorical variables were compared using Fisher’s exact test, and continuous variables were compared using the Mann-Whitney U test or Student’s *t* test, depending on the data distribution. The results are presented as odds ratios (ORs) with 95% confidence intervals (CIs). A multivariate logistic regression analysis was performed to explore factors potentially associated with the occurrence of BDD. The diameter of the endoscope working channel, maximum balloon diameter, and number of EBD attempts within the same session were included as covariates, based on clinical relevance and procedural considerations.

For the sensitivity analysis, we performed a mixed-effects logistic regression analysis with patient ID included as a random effect to account for the potential within-patient correlation arising from multiple EBD sessions performed in the same patient. We also performed a sensitivity analysis restricted to the first EBD session for each patient.

To assess the interobserver reliability of the BDD assessment, an independent coauthor blinded to the original assessment independently re-evaluated a random sample of 100 EBD sessions for the presence or absence of BDD. Interobserver agreement was assessed using Cohen’s kappa coefficient.

We reported 95% two-sided CIs as an informal measure of uncertainty and followed the recommendations of the American Statistical Association to avoid the use of terms such as “statistically significant” [9]. All the analyses were performed using Stata version 18.0 (StataCorp).

## Results

### Patient and Procedure Characteristics

During the study period, 351 EBD sessions were conducted for gastrointestinal strictures associated with CD. Among them, 32 sessions performed using conventional colonoscopy were excluded because the present study focused on EBD procedures performed using balloon-assisted enteroscopes. Finally, 319 EBD sessions were included in the analysis. The 319 EBD sessions involved 55 unique patients. A flowchart of EBD session inclusion and exclusion is presented in Figure 1. Patient characteristics and endoscopic procedural factors are summarized in Table 1. Regarding procedural characteristics, the median number of EBD attempts per session was 2 (interquartile range, 1–3), and 182 sessions (57.1%) included ≥2 EBD attempts within the same session.

**Fig. 1.**
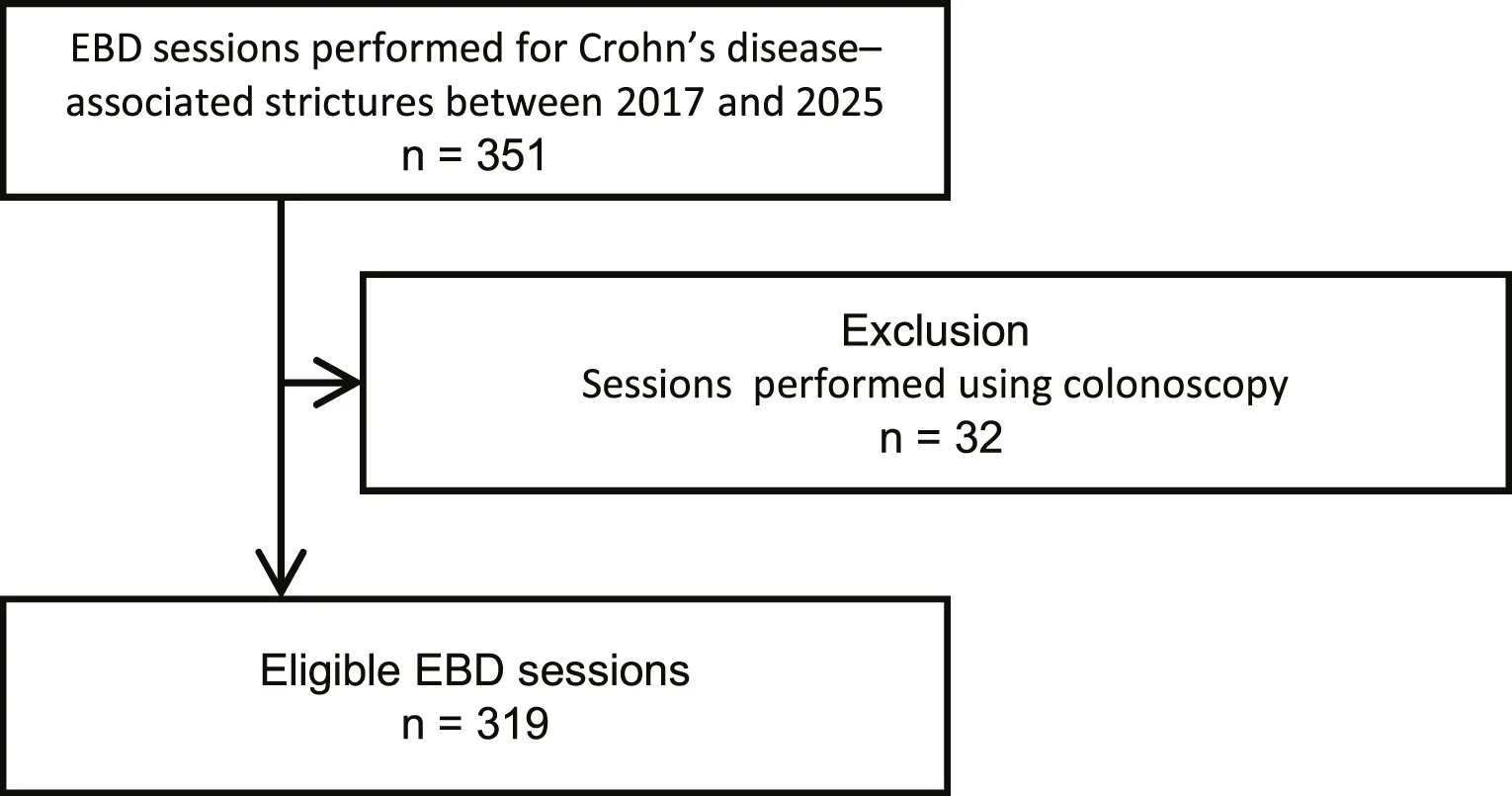
Flow diagram of endoscopic balloon dilation (EBD) session selection included in the analysis.

**Table 1.** Baseline characteristics of EBD sessions

Demographics	*N* = 319
Male, *n* (%)	233 (73.0)
Age, years (median, IQR)	50 (44–54)
Disease duration, years (median, IQR)	21 (14–26)
Disease location, *n* (%)	
L1	124 (38.9)
L3	195 (61.1)
Concomitant medications, *n* (%)	
5-aminosalicylic acid	293 (91.8)
Thiopurine	101 (31.7)
Anti-TNF-α agents	181 (56.7)
Ustekinumab	41 (12.9)
Anti-IL-23 p19 agents	5 (1.6)
Vedolizumab	1 (0.3)
Balloon catheter type, *n* (%)	
EZDilate (Olympus Medical Systems, Tokyo, Japan)	224 (70.2)
Elation 5-Stage Fixed-Wire Dilation Balloon (Merit Medical Systems, Inc., South Jordan, UT, USA)	87 (27.3)
Hercules 3 Stage Wire-Guided Balloon (Cook Medical, Bloomington, IN, USA)	8 (2.5)
Working channel diameter, *n (%)*	
2.8 mm	50 (15.7)
3.2 mm	269 (84.3)
Scope length, *n* (%)	
Short type	25 (7.8)
Long type	294 (92.2)
History of prior intestinal surgery, *n* (%)	291 (91.2)
Number of EBD attempts per session, *n* (%)	
1	137 (42.9)
2	113 (35.4)
≥3	69 (21.6)
Maximum balloon diameter, *n* (%)	
<20 mm	165 (51.7)
≥20 mm	154 (48.3)
Number of strictures, *n* (%)	
1	53 (16.6)
2	84 (26.3)
≥3	182 (57.1)
Stricture location, *n* (%)	
Ileocecal region	35 (11.0)
Ileocolonic anastomosis	173 (54.2)
Small intestine	310 (97.2)
Anus	35 (11.0)

Baseline patient characteristics and endoscopic procedural factors in the analyzed EBD sessions. Data are presented as medians with interquartile ranges (IQRs) for continuous variables and as numbers with percentages for categorical variables. The unit of analysis was the individual EBD session. An EBD attempt was defined as a single balloon dilation procedure, and the number of EBD attempts represented the total number of balloon dilations performed within a single session. The structural locations were not mutually exclusive.

EBD, endoscopic balloon dilation.

The diameter of the working channel was 2.8 mm in 50 sessions (15.7%) and 3.2 mm in 269 sessions (84.3%). Short enteroscopes were used in 25 sessions (7.8%), whereas long enteroscopes were used in 294 sessions (92.2%). A maximum balloon dilation diameter of ≥20 mm was used in 154 sessions (48.3%).

### Incidence and Types of BDD

The incidence and types of BDD are shown in Figure 2. BDD was observed in 33 sessions (10.3%). There were 12 cases of difficulty in balloon withdrawal, 24 cases of difficulty in balloon insertion, and 6 cases of balloon damage. Some EBD sessions were complicated by > 1 type of BDD; therefore, the total number of BDD events exceeded the number of sessions. For timing analysis, each session was counted once, according to the first occurrence of BDD within that session. When the timing of BDD occurrence within a session was examined, 6 events occurred during the first EBD attempt (6 of 319 attempts), whereas 27 occurred during the second and subsequent EBD attempts (27 of 271 attempts) within the same session. Notably, difficulty in balloon insertion occurred in 3 events during the first EBD attempt, compared with 21 events during the second and subsequent EBD attempts.

**Fig. 2.**
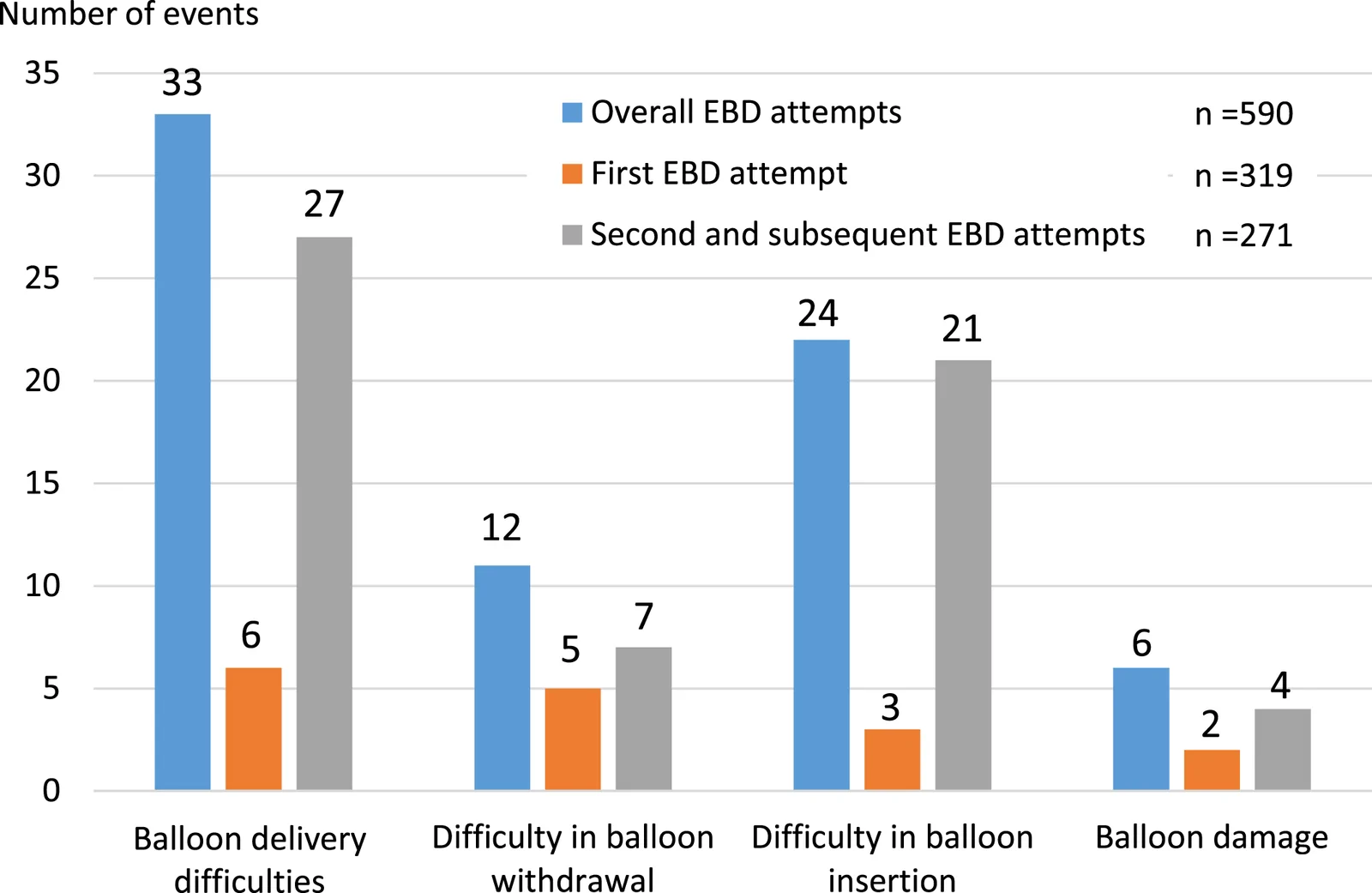
Incidence and timing of balloon delivery difficulties (BDD) during endoscopic balloon dilation (EBD). The overall incidence and types of BDD observed during EBD sessions are shown. The distribution of BDD according to the number of EBD attempts within a session is presented and stratified into first and second or subsequent attempts. BDD included difficulty in balloon withdrawal, balloon insertion, and balloon damage. Each EBD attempt is treated as an independent opportunity for balloon delivery-related events.

### Factors Associated with the Occurrence of BDD

The results of the univariate analyses examining the factors associated with the occurrence of BDD are shown in Table 2. In univariable analyses, a higher number of EBD attempts within the same session (OR, 2.56; 95% CI, 1.07–6.80; *p* = 0.025) and the use of an endoscope with a 2.8-mm working channel, compared with a 3.2 mm working channel (OR, 3.13; 95% CI, 1.28–7.69; *p* = 0.009), were associated with the occurrence of BDD. In multivariable logistic regression analysis, the number of EBD attempts within the same session (adjusted OR, 2.54; 95% CI, 1.01–5.88; *p* = 0.029) and the use of a 2.8-mm working channel (adjusted OR, 2.94; 95% CI, 1.28–6.67; *p* = 0.011) were independently associated with the occurrence of BDD.

**Table 2.** Factors associated with balloon delivery difficulties

​	Total	BDD	Univariable analysis		
	*N*	*N* (%)	OR	95% CI	*p* value
Number of EBD attempts per session					
1	137	8 (5.8)	Reference	​	​
≥2	182	25 (13.7)	2.56	1.07–6.80	0.025
Working channel diameter					
3.2 mm	269	22 (8.2)	Reference	​	​
2.8 mm	50	11 (22.0)	3.13	1.28–7.69	0.009
Maximum balloon diameter					
<20 mm	165	18 (10.9)	Reference	​	​
≥20 mm	154	15 (9.7)	0.88	0.40–1.93	0.85
Scope length					
Long type	294	32 (11.0)	Reference	​	​
Short type	25	1 (4.0)	0.34	0.01–2.25	0.49
Ileocolonic anastomosis stricture					
Yes	173	18 (10.4)	Reference	​	​
No	146	15 (10.3)	1.01	0.46–2.25	1.00
Ileocecal region stricture					
Yes	35	2 (5.7)	Reference	​	​
No	284	31 (10.9)	0.49	0.05–2.11	0.56

BDD, balloon delivery difficulty; EBD, endoscopic balloon dilation; OR, odds ratio; CI, confidence interval.

Univariable logistic regression analysis of factors associated with BDD during EBD. ORs with 95% CIs are shown.

To evaluate the potential influence of balloon catheter type, we reviewed the distribution of balloon catheters used during EBD. EZDilate (Olympus Medical Systems) was used in 224 sessions (70.2%), ELATION (Merit Medical Systems) in 87 sessions (27.3%), and Hercules 3 Stage (Cook Medical) in 8 sessions (2.5%). In an exploratory multivariable analysis additionally adjusting for balloon type, balloon type was not associated with BDD occurrence (OR, 0.70; 95% CI, 0.41–1.18; *p* = 0.180), and the overall direction of the associations between working channel diameter, number of EBD attempts, and BDD remained unchanged. A sensitivity analysis restricted to sessions using EZDilate also demonstrated a persistent association between the working channel diameter and BDD (OR, 43.5; 95% CI, 3.83–500.0; *p* = 0.002).

Furthermore, the incidence of BDD varied numerically throughout the study period. These findings are summarized in online supplementary Table 1 (for all online suppl. material, see https://doi.org/10.1159/iid/adeag002). In the early study period (up to 2019), only enteroscopes with a 2.8-mm working channel were used, during which BDD occurred in 10/39 sessions (25.6%).

### Procedural Consequences of BDD

Among the sessions with BDD, deep insertion could not be completed in 16 sessions (48.5%), and planned dilation was abandoned in 14 sessions (42.4%). An additional balloon catheter was required in 23 sessions (69.7%).

### Adverse Events

During the study period, EBD-related perforation occurred in 1 session (0.3%), and EBD-related bleeding occurred in 19 sessions (5.6%). No other major procedure-related adverse events were observed.

### Interobserver Reliability of BDD Assessment

In the interobserver reliability analysis of 100 randomly selected EBD sessions, the overall agreement for BDD assessment was 93.0%, with a Cohen’s κ coefficient of 0.63, indicating substantial interobserver agreement.

## Discussion

In this retrospective single-center study, BDD occurred in approximately 10% of EBD sessions for CD-associated strictures and was more frequently observed during repeated dilation attempts within the same session. Difficulty in balloon insertion was the most common type of BDD. To our knowledge, this is the first study to systematically evaluate the incidence, patterns, and risk factors of BDDs during EBD in CD-associated strictures. Although BDD did not directly result in major adverse events, it frequently necessitated additional procedural maneuvers, including failure of deep insertion, abandonment of planned dilation, and the use of additional balloon catheters, highlighting its practical clinical impact.

Although several studies have reported the overall safety and efficacy of EBD for CD-associated strictures [10, 11], technical issues related to balloon delivery have not been specifically addressed as previous reports have mainly focused on major adverse events such as perforation or bleeding. In this context, this study adds to the existing literature by highlighting balloon delivery as a distinct technical component of EBD.

While BDD increased with the number of EBD attempts within the same session, this finding may reflect the increased number of balloon delivery maneuvers. However, the pattern of BDD occurrence was not uniform when individual complication types were examined. Difficulty in balloon insertion occurred predominantly during the second and subsequent EBD attempts, whereas difficulty in balloon withdrawal and damage were observed even during the first attempt. These findings suggest that once balloon dilation is performed, technical vulnerabilities related to balloon delivery may already be introduced, potentially due to incomplete balloon deflation or structural deformation of the balloon catheter.

Balloon delivery is a potential procedural bottleneck in EBD. Several mechanisms can explain the occurrence of BDD. First, a balloon used for dilation may not be completely deflated, and residual deformation may increase resistance during passage through the working channel. Second, although the balloon catheter is straightened before use, it may become curved after dilatation (Fig. 3). This change in shape is more likely to occur when angulated strictures are dilated. When the same balloon is used repeatedly for multiple dilations within a single session, these factors may compound, thereby increasing the likelihood of BDD. Although the present study focused on CD-associated strictures, the mechanisms underlying BDD are unlikely to be specific to CD. Difficulties in balloon delivery may also occur during endoscopic dilation of other gastrointestinal strictures. Further studies are required to determine whether similar technical challenges exist in non-IBD populations.

**Fig. 3.**
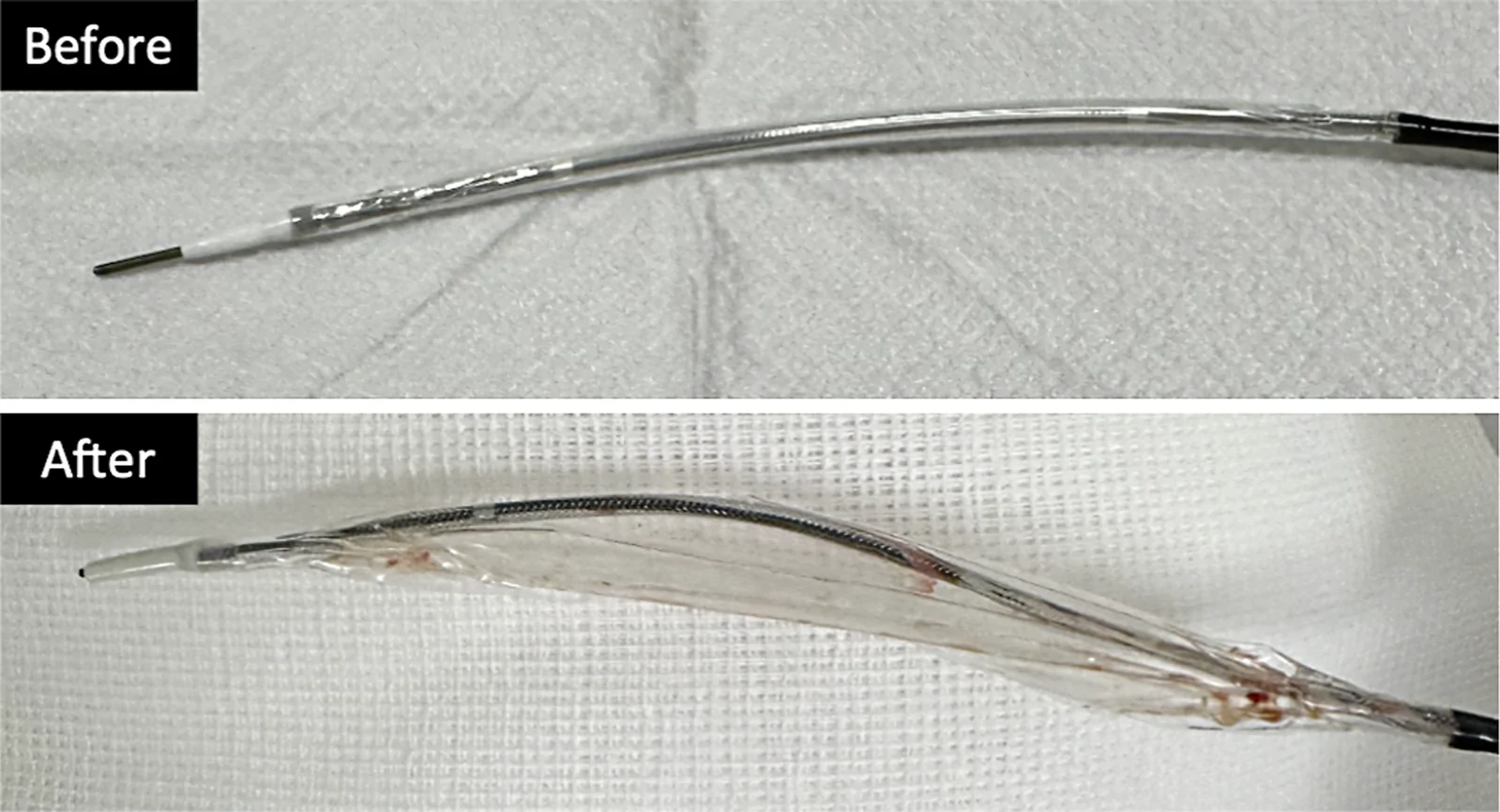
Representative images of a balloon catheter before and after dilation. The catheter appears straight before use (before), whereas after dilation (after), the catheter shaft becomes curved, and the balloon may not fully return to its original shape after deflation.

The findings of this study have several clinically relevant implications for procedural strategies during EBD. In particular, when multiple dilations are anticipated within the same session, endoscopists should be aware of the increased risk of BDD, especially during balloon insertion. BDD may lead to prolonged procedure time or even premature termination of EBD. Consequently, these events may compromise procedural success, potentially resulting in the need for surgical intervention or inadequate evaluation of more proximal lesions due to limited scope advancement. Therefore, BDD can be considered a clinically relevant issue that may impact the overall quality of EBD. Based on our findings, endoscopists should recognize that BDD is not uncommon during EBD, particularly when repeated dilation attempts are required and when enteroscopes with a 2.8-mm working channel are used. Awareness of this risk may facilitate procedural planning and help avoid interruptions in the evaluation and treatment of more proximal lesions in patients with multiple strictures. Future refinements in procedural techniques and device design focusing on balloon delivery may further improve the safety, procedural efficiency, and technical reliability of EBD.

This study had several limitations. First, this was a single-center, retrospective study. As a result, relatively minor BDD events, such as prolonged difficulty during balloon insertion, may not have been consistently documented in medical records, potentially leading to an underestimation of the true incidence of BDD. Second, direct quantitative assessment of BDDs is inherently challenging. Because this was a retrospective study based on routine endoscopy reports, objective operational measures, such as procedure time, number of additional insertions or withdrawal attempts, or traction force, were not available. Therefore, surrogate outcomes, including difficulty in balloon withdrawal, difficulty in balloon insertion, and balloon damage, were used. Third, certain factors that may be associated with BDD but are difficult to quantify, such as the degree of stricture angulation, could not be adequately evaluated. Furthermore, the inflammatory or fibrotic nature of individual strictures was not systematically assessed, and reliable retrospective classification was difficult because inflammatory and fibrotic components frequently coexist in CD strictures. Finally, operator-related factors, including endoscopist experience and procedural volume, were not systematically available in this retrospective study and, therefore, could not be evaluated, although these factors may potentially influence the occurrence of BDD.

In conclusion, this study demonstrated that BDD occurred in approximately 10% of EBD sessions and was more likely during repeated dilation attempts. Awareness of these technical issues may help endoscopists optimize procedural strategies and improve the efficiency and technical reliability of EBD in clinical practice.

## Acknowledgments

We would like to express our sincere gratitude to the staff of the Endoscopy Center at Yokohama Municipal Citizen’s Hospital for their cooperation in performing endoscopic examinations and collecting clinical data. We also thank Editage (https://www.editage.jp) for English-language editing. Declaration of generative AI and AI-assisted technologies in the manuscript preparation process: during the preparation of this manuscript, we used ChatGPT (OpenAI) to assist with language editing and refinement. After using this tool, the authors reviewed and edited the content as required and took full responsibility for the content of the published article.

## Statement of Ethics

The study protocol was reviewed and approved by the Yokohama Municipal Citizen’s Hospital (Approval No. 26-02-04). Owing to the retrospective study design and the use of clinical data obtained during routine clinical practice, the requirement for informed consent was waived, and an opt-out approach was adopted. This study was conducted in accordance with the principles of the Declaration of Helsinki.

## Conflict of Interest Statement

The authors have no conflicts of interest to declare.

## Funding Sources

This study was not supported by any sponsor or funding.

## Author Contributions

Conceptualization, data curation, formal analysis, writing – original draft, software, visualization, methodology, and project administration: T.F.; investigation, validation, writing – review and editing, and approval of final manuscript: T.F., Y.M., K.M., Y.H., Y.T., S.I., S.N., Y.K., and Y.F.; resources: T.F. and Y.M.; and supervision: Y.M. and Y.K.

## Data Availability

The data supporting the findings of this study are not publicly available because of the presence of potentially identifiable or sensitive patient information. However, the data may be made available upon reasonable request, subject to review and approval, provided that the researchers meet the criteria for access to confidential data. Further inquiries can be directed to the corresponding authors.

## References

[1] Cosnes J , Cattan S, Blain A, Beaugerie L, Carbonnel F, Parc R, . Long-term evolution of disease behavior of crohn’s disease. Inflamm Bowel Dis. 2002;8(4):244–50. 10.1097/00054725-200207000-00002 12131607

[2] Freeman HJ . Natural history and clinical behavior of crohn’s disease extending beyond two decades. J Clin Gastroenterol. 2003;37(3):216–9. 10.1097/00004836-200309000-00005 12960719

[3] Farmer RG , Whelan G, Fazio VW. Long-term follow-up of patients with Crohn’s disease. Gastroenterology. 1985;88(6):1818–25. 10.1016/0016-5085(85)90006-x 3922845

[4] Bettenworth D , Gustavsson A, Atreja A, Lopez R, Tysk C, Van Assche G, . A pooled analysis of efficacy, safety, and long-term outcome of endoscopic balloon dilation therapy for patients with stricturing crohnʼs disease. Inflamm Bowel Dis. 2017;23(1):133–42. 10.1097/MIB.0000000000000988 28002130

[5] Adamina M , Minozzi S, Warusavitarne J, Buskens CJ, Chaparro M, Verstockt B, . ECCO guidelines on therapeutics in crohn’s disease: surgical treatment. J Crohns Colitis. 2024;18(10):1556–82. 10.1093/ecco-jcc/jjae089 38878002

[6] Bettenworth D , Bokemeyer A, Kou L, Lopez R, Bena JF, El Ouali S, . Systematic review with meta‐analysis: efficacy of balloon‐assisted enteroscopy for dilation of small bowel Crohn’s disease strictures. Aliment Pharmacol Ther. 2020;52(7):1104–16. 10.1111/apt.16049 PMC8052861 32813282

[7] Bamba S , Sakemi R, Fujii T, Takeda T, Fujioka S, Takenaka K, . A nationwide, multi-center, retrospective study of symptomatic small bowel stricture in patients with Crohn’s disease. J Gastroenterol. 2020;55(6):615–26. 10.1007/s00535-020-01670-2 31989252

[8] Hirai F , Beppu T, Takatsu N, Yano Y, Ninomiya K, Ono Y, . Long‐term outcome of endoscopic balloon dilation for small bowel strictures in patients with C rohn’s disease. Dig Endosc. 2014;26(4):545–51. 10.1111/den.12236 24528293

[9] Wasserstein RL , Lazar NA. The ASA statement on *p* -Values: Context, process, and purpose. Am Stat. 2016;70(2):129–33.

[10] Klag T , Wehkamp J, Goetz M. Endoscopic balloon dilation for crohn’s disease-associated strictures. Clin Endosc. 2017;50(5):429–36. 10.5946/ce.2017.147 PMC5642070 29017297

[11] Morar PS , Faiz O, Warusavitarne J, Brown S, Cohen R, Hind D, . Systematic review with meta-analysis: endoscopic balloon dilatation for Crohn’s disease strictures. Aliment Pharmacol Ther. 2015;42(10):1137–48. 10.1111/apt.13388 26358739

